# Health-related quality of life in cardiac sarcoidosis: a systematic review

**DOI:** 10.1093/ehjopen/oead009

**Published:** 2023-02-18

**Authors:** Juan Carlos Quijano-Campos, Neha Sekhri, Muhunthan Thillai, Julie Sanders

**Affiliations:** William Harvey Research Institute, Queen Mary University of London, Charterhouse Square, London EC1M 6BQ, UK; St Bartholomew’s Hospital, Barts Health NHS Trust, West Smithfield, London EC1A 7DN, UK; Research & Development, Royal Papworth Hospital NHS Foundation Trust, Papworth Road, Cambridge CB2 0AY, UK; Barts Heart Centre, St Bartholomew’s Hospital, Barts Health NHS Trust, West Smithfield, London EC1A 7BE, UK; Interstitial Lung Disease Unit, Royal Papworth Hospital NHS Foundation Trust, Papworth Road, Cambridge CB2 0AY, UK; Department of Medicine, School of Clinical Medicine, University of Cambridge, Cambridge CB2 0SP, UK; William Harvey Research Institute, Queen Mary University of London, Charterhouse Square, London EC1M 6BQ, UK; St Bartholomew’s Hospital, Barts Health NHS Trust, West Smithfield, London EC1A 7DN, UK

**Keywords:** Cardiac sarcoidosis, Health-related quality of life (HRQoL), Patient-reported outcome measures (PROMS), Systematic review

## Abstract

People living with cardiac sarcoidosis (CS) are likely to have worse clinical outcomes and greater impairment on health-related quality of life (HRQoL) than other sarcoidosis manifestations. CS can result in a constellation of intrusive symptoms (such as palpitations, dizziness, syncope/pre-syncope, chest pain, dyspnoea, orthopnoea, or peripheral oedema) and/or life-threatening episodes, requiring consideration of invasive cardiac procedures for diagnosis and for the management of acute events. Additionally, the presence of multisystemic involvement and persistent non-specific sarcoidosis symptoms negatively affect HRQoL. A systematic review was undertaken to explore the impact of CS on HRQoL in adults with CS. Multiple bibliographic databases were searched for studies with HRQoL as primary or secondary outcomes in CS (PROSPERO registration: CRD42019119752). Data extraction and quality assessments were undertaken independently by two authors. From the initial 1609 identified records, only 11 studies included CS patients but none specifically reported HRQoL scores for CS patients. The average representation of CS patients was 14.5% within these cohorts (range 2–22%). The majority (73%) was conducted in single-centre tertiary care settings, and only one study (9%) included longitudinal HRQoL data. CS patients were among those sarcoidosis patients with impaired HRQoL and worse outcomes, requiring higher doses of sarcoidosis-specific therapy which contribute to further deterioration of HRQoL. Sarcoidosis studies do not incorporate stratified HRQoL scores for CS patients. While there is a need for longitudinal and multicentre studies assessing HRQoL outcomes in CS cohorts, the development of CS-specific tools is also needed.

## Graphical Abstract

**Graphical abstract:**
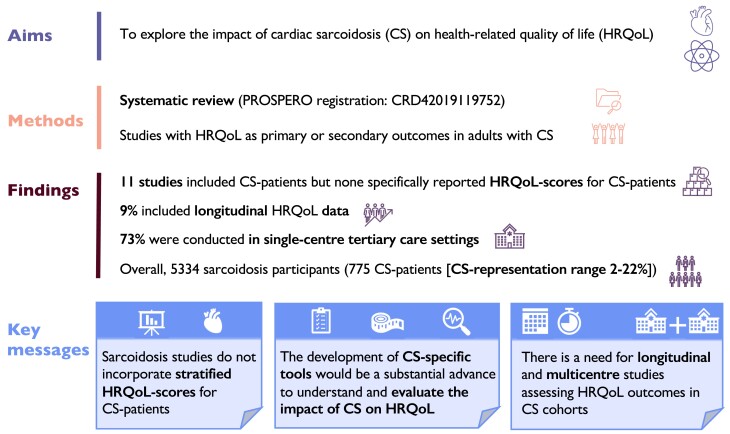

## Introduction

Cardiac sarcoidosis (CS) is a rare and potentially life-threatening condition characterized by formation of non-caseating granulomas in the heart, which could be detected as part of multi-organ sarcoidosis involvement or may occur in isolation.^1^ The most feared manifestation of CS is sudden cardiac death,^2^ which may be due to ventricular arrhythmias or conduction abnormalities causing complete heart block.^3,4^ Previous studies have shown that CS is clinically manifested in 5% of patients with sarcoidosis.^5,6^ However, imaging and autopsy studies estimate the prevalence of cardiac involvement ∼20–35%.^7–11^ Diagnosis of CS can be challenging as patients can be asymptomatic or present with a constellation of intrusive symptoms such as palpitations, dizziness, syncope/pre-syncope, chest pain, dyspnoea, orthopnoea, or peripheral oedema.^11–14^ Management of CS requires careful consideration of cardiac medication and invasive cardiac procedures [e.g. endomyocardial biopsy, arrhythmia ablation, implantation of cardiac devices (loop recorder, pacemaker, and/or defibrillator), and in very rare cases heart transplant] in addition to higher doses of immunosuppressive medication with associated potential side effects. Therefore, some people living with CS face additional burden of cardiac symptoms and uncertainties compared with those without cardiac involvement.

Health-related quality of life (HRQoL) is a multi-dimensional concept that reflects the impact of a health condition on involvement and fulfilment of important life areas.^15^ It includes general health, physical symptoms and toxicity, and existential issues and domains related to physical, cognitive, emotional, sexual, social, and role functioning.^16^ Incorporating HRQoL assessments in routine practice facilitates the patient–clinician communication and enables healthcare professionals to identify moderate-to-severe problems related to HRQoL.^17^ The use of patient-reported outcome measures (PROMs) to assess HRQoL has become increasingly important in the management of patients with cardiac diseases. Generic and disease-specific PROMs are essential tools to measure the quality of cardiovascular care, capturing ‘what truly matters to our patients’.^18^ The use of PROMS provides several benefits for individual patient care. For example, PROMS can facilitate patient-centred care, promote early identification of health issues, and improve outcomes.^19^ Moreover, PROMS can reduce unnecessary clinical appointments for stable patients, optimizing the use of limited healthcare resources.^20^ Equally, PROMS have shown benefits for clinical teams, service managers, commissioners, regulators, and policy makers.^21^ Currently, NHS Digital is using PROMS to calculate health gains on patients undergoing surgical treatment (hip or knee replacement).^22^

The burden of sarcoidosis could affect somatic, psychosocial, and economic aspects of life.^23,24^ Considering that the indications for treating sarcoidosis are aimed either to ‘avoid danger or to improve HRQoL’,^25^ this approach is particularly pertinent in CS. Firstly, CS is potentially dangerous and, without treatment, could lead to progressive cardiac dysfunction or sudden cardiac death.^2,26^ Therefore, prompt initiation of systemic treatment is recommended in patients with CS,^27^ which includes steroid and steroid-sparing medication. However, this therapy may itself worsen the overall HRQoL due to side effects and need for frequent monitoring visits for avoiding and early identification of drug toxicity. Secondly, sarcoidosis patients consider HRQoL as the main treatment outcome.^28^ In addition to organ-related symptoms, sarcoidosis patients may suffer from a wide range of persistent non-specific (‘parasarcoidosis’) symptoms which could impact on HRQoL, such as fatigue, weight reduction, pain, general weakness, exercise limitation, everyday cognitive impairment, poor subjective sleep quality, and depressive symptoms.^29–31^ It has been suggested that the impact on HRQoL is greater in sarcoidosis patients with cardiac involvement.^32^ However, the sarcoidosis-specific HRQoL PROMs were developed without representation from patients with CS, and there is a lack of information related to the impact of CS in HRQoL which limits clinical decisions and future research focus. Thus, we sought to undertake a systematic review to identify and synthetize existing studies exploring the impact of CS on HRQoL in adults.

## Methods

### Protocol and registration

This review was registered on PROSPERO, an international prospective register of systematic review (February 2019, reference CRD42019119752) and is reported in accordance with the Preferred Reporting Items for Systematic Reviews and Meta-Analyses (PRISMA) guidelines.^33^

### Eligibility criteria

All studies which included HRQoL as primary or secondary outcomes in adults with a diagnosis of cardiac sarcoidosis were eligible for inclusion. Studies were included if they met the following inclusion and exclusion criteria: *population*, adults (aged ≥18 years) living with cardiac sarcoidosis, and *outcome*, HRQoL as primary or secondary outcome but excluding quantitative studies and those using a non-validated HRQoL tool. Studies were not excluded on the basis of publication date or quality assessment but were required to be available in English language.

### Information sources

The search was conducted on 24 April 2022 including the following electronic databases: MEDLINE, Embase, Cumulated Index of Nursing and Allied Health Literature (CINAHL), British Nursing Index (BNI), Allied and Complementary Medicine Database (AMED), PsycINFO, Applied Social Sciences Index and Abstracts (ASSIA), Cochrane Library, Cuiden plus, Campbell Collaboration, Joanna Briggs Institute (JBI), Scopus, and Web of Science. Due to the high specificity of a rare condition such as cardiac sarcoidosis, supplementary search strategies were undertaken covering grey literature databases [OpenGrey, ProQuest, GreySource, Electronic Theses Online Service (EThOS), Touro College, Social Care Online, and British Education Index], contacting authors for unpublished studies, systematic reference list checking, citation searching from key studies, and publication list from key authors in the field.

### Search strategy

The search syntax was adapted for each database using title/abstract text terms paired with (majored) exploded Medical Subject Headings (MeSH) terms, or equivalent, and a pearl growing technique with different combinations: ((*Mesh descriptor*: [Sarcoidosis] *explode all trees* AND cardiac) OR (cardiac ADJ3 sarcoid*) OR (heart ADJ3 sarcoid*).ti, ab) AND (*Mesh descriptor*: [Quality Of Life] *explode all trees* OR *Mesh descriptor*: [Fatigue] *explode all trees* OR (“quality of life” OR qol OR “health status” OR wellbeing OR satisfaction OR improvement* OR fatigue OR energy OR tiredness OR exhaustion OR anxi* OR burden OR exercise OR cognitive OR depressi* OR driv* OR pain* OR panic OR rehab*).ti, ab). No date, setting, or context restrictions were applied. A detailed search string for each database is listed in the Supplementary material online, *Table S1*.

### Study selection

After removal of duplicates, the review team (J.C.Q-C., J.S., M.T., and N.S.) completed two rounds of screening. First, titles and abstracts from the initial search (electronic databases) were independently screened, followed by full-text review of potentially relevant records from the first screening. All screening was undertaken independently by the first reviewer (J.C.Q-C.) and a second reviewer (J.S., M.T., and N.S.). Discordances were resolved by consensus. Due to the high specificity of cardiac sarcoidosis, a second stage of study selection included supplementary searching strategies to optimize identification of potential studies, following the above screening process. A detailed search string for each database is listed in the Supplementary material online, *Table S1*.

### Data extraction (data collection process and data items)

Data from eligible studies were extracted independently by two reviewers (J.C.Q-C. first review and second reviewers one of J.S., M.T., and N.S.) on to a standardized proforma, with discordances resolved by consensus. Extracted data included primary author, publication date, settings and country of study, study recruitment dates, study design, population and sample size, eligibility criteria, PROMs used and assessment timepoints, and socio-demographic and clinical characteristics. HRQoL information included results for domains related to generic or sarcoidosis-specific HRQoL tools at each timepoint, adjusted HRQoL results for cardiac involvement, and any specific subgroups with significant differences for HRQoL.

### Quality assessment and risk of bias

The quality assessment was performed in parallel with the data extraction process. The relevant Critical Appraisal Skills Programme (CASP) template was used by two reviewers.^34^

The CASP was subsequently aggregated to develop a score for each publication as a percentage of the number of met criteria out of the number of applicable. These quality assessment scores were organized into categorized previously reported.^35,36^ Studies rated above 80% were considered high quality, 60–79% were moderate quality, and lower than 59% were appraised as poor quality. No studies were excluded on the basis of the quality assessment to avoid the potential exclusion of insightful studies. Graphs were generated to summarize the CASP checklists and the risk of bias summary.^37^ Analysis results were summarized using descriptive statistics, tables, and narrative synthesis, as appropriate. Interpretation of the analysis was discussed and agreed by all reviewers.

### Patient and public involvement

The patient and public involvement group (five members with cardiac sarcoidosis diagnosis) helped to inform the scope of the research questions and contributed to the PROSPERO protocol.

## Results

### Study selection

**Figure 1:**
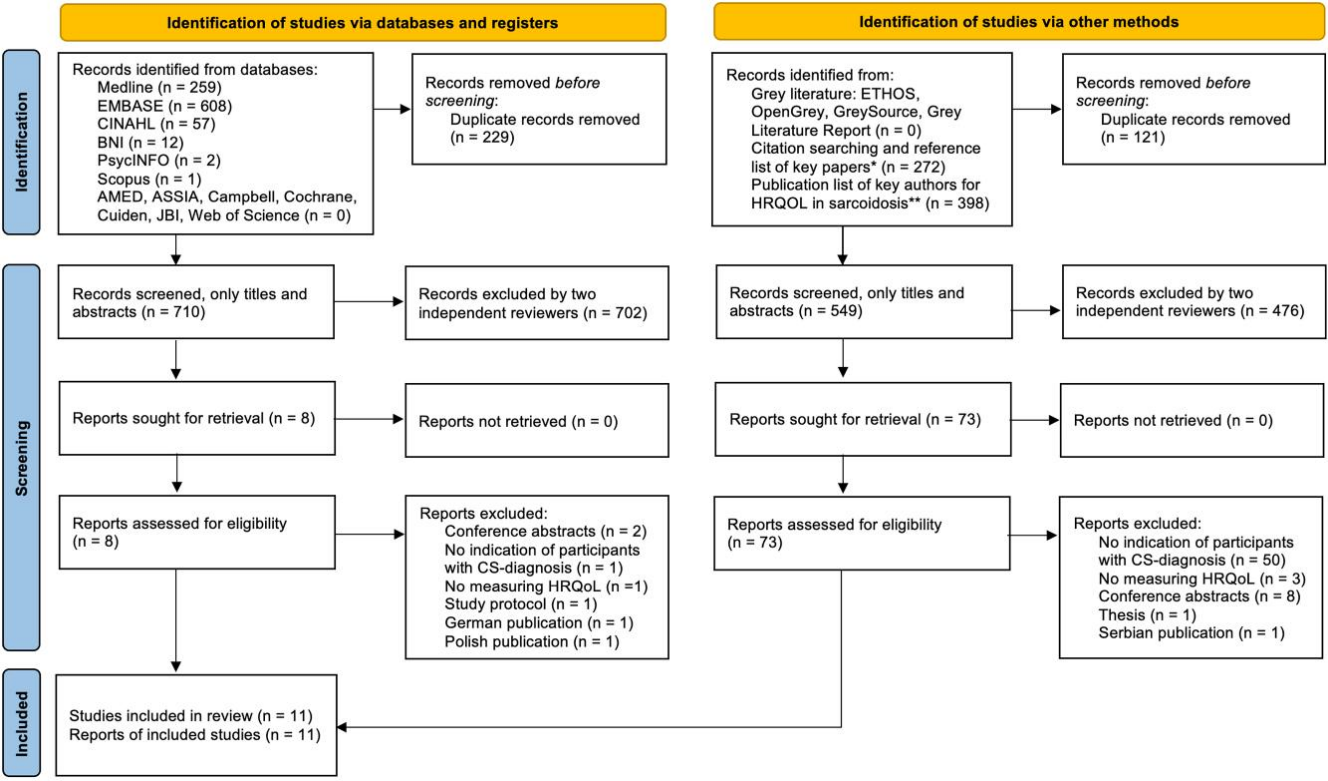
Adapted PRISMA flow diagram.^33^ The PRISMA diagram details our search and selection process applied during the overview. *The four ‘key papers’ included three peer-reviewed publications for the sarcoidosis-specific HRQoL questionnaires (SHQ, KSQ, and SAT) and one study which was selected for inclusion in the review (identified via electronic databases). **This supplementary searching technique included the following seven ‘key authors’ in HRQoL for sarcoidosis: Baughman, R.P.; Birring, S.S.; Drent, M.; Gozdenovic, B.S; Judson, M.A.; Wells, A.U.; and Wijsenbeek, M.S. AMED, allied and complementary medicine database; ASSIA, applied social sciences index and abstracts; BNI, British nursing index; CINAHL, cumulated index of nursing and allied health literature; CS, cardiac sarcoidosis; EThOS, electronic theses online service; HRQoL, health-related quality of life; JBI, Joanna Briggs Institute; KSQ, King’s sarcoidosis questionnaire; SAT, sarcoidosis assessment tool; SHQ, sarcoidosis health questionnaire.

*Figure 1* shows the adapted PRISMA flowchart.^33^ In brief, a total of 1609 potentially relevant records were identified. Following title and abstract review, 81 went on to full-text review resulting in 11 studies being included for analysis.^38–48^

### Study characteristics

*Table 1* summarizes the study characteristics, and *Table 2* describes clinical information and the HRQoL results for the 11 included studies.

**Table 1:** Study characteristics and level of evidence of each included study (*n* = 11)

Study	Country	Design	Data collection period	Diagnostic criteria tool used (year)	Population	Sample size (*n*); cardiac sarcoidosis (%)	HRQoL PROM used	HRQoL assessment timepoints	Level of evidence, CASP (%)
Bourbonnais *et al*. (2010)^38^	USA	Observational cohort	December 2005–May 2009	ATS/ERS/WASOG (1999)^27^	221 patients with tissue-proven diagnosis of sarcoidosis	*n* = 221, 5%	SF-36 SHQ	Single	Moderate (64%)
Bourbonnais *et al*. (2012)^39^	USA	Observational cohort	December 2005–May 2008	ATS/ERS/WASOG (1999)^27^	162 patients with a tissue-proven diagnosis of sarcoidosis	*n* = 162, 4%	SF-36 SHQ	Single	Poor (36%)
Elfferich *et al*. (2011)^40^	The Netherlands	Cross-sectional	November 2007	ATS/ERS/WASOG (1999)^49^	Set of questionnaires sent to 68 IPF and 588 sarcoidosis patients	*n* = 441, 8%	WHOQOL BREF	Single	Poor (45%)
Frye *et al*. (2021)^41^	Germany	Observational cohort	February 2016–October 2016^a^	ATS/ERS/WASOG (1999)^27^	200 consecutive sarcoidosis patients	*n* = 200 3%	KSQ	Single	Moderate (64%)
Gvozdenovic *et al*. (2008)^42^	Serbia	Cross-sectional	October 2006–March 2007	ATS/ERS/WASOG (1999)^49^	81 consecutive patients with biopsy-proven pulmonary sarcoidosis	*n* = 81, 2%	15-D SGRQ	Single	Poor (55%)
Harper *et al*. (2020)^43^	USA	Observational cohort, web-based platform	June 2014–December 2016	Self-reported, patient opt-in, FSR-SARC web^50^	2630 respondents to the FSR-SARC	*n* = 2318, 21%	SHQ	Single	Poor (45%)
Judson *et al*. (2019)^44^	USA	Retrospective analysis	May 2011–February 2018	ATS/ERS/WASOG (1999)^49^ and ACCESS (1999)^51^; from August 2014 WASOG (2014)^52^	660 consecutive sarcoidosis patients in the local registry	*n* = 655, 8%	SAT	Single^b^	Poor (38%)
Judson *et al*. (2022)^45^	USA	Observational cohort, prospective longitudinal	April 2017–December 2020^c^	ATS/ERS/WASOG (1999)^49^ and ATS(2020)^53^	332 sarcoidosis patients enrolled into the OSAP	*n* = 332, 16%	KSQ SAT SGRQ	Baseline and 6-month	Moderate (62%)
MihailovićVučinić *et al*. (2016)^46^	Serbia	Cross-sectional	Not stated	ACCESS(19992003)^51,54,55^	346 patients with biopsy-confirmed sarcoidosis	*n* = 346, 7%	Patient-VAS SHQ	Single	Poor (45%)
Obi *et al*. (2022)^47^	USA and UK	Longitudinal database	January 2017–December 2019^d^	WASOG (2014)^52^ and ATS (2020)^53^	465 patients enrolled into the ReAS	*n* = 456, 17%	KSQ SGRQ	Baseline, 12-month, 24-month and 36-month	Moderate (69%)
Tanizawa *et al*. (2019)^48^	Japan	Observational cohort study	June 2009 December 2009	ATS/ERS/WASOG (1999)^49^	122 consecutive patients with biopsy-supported sarcoidosis	*n* = 122, 22%	SF-36 SHQ SGRQ	Single	Moderate (77%)

15D, 15-dimensional measure of HRQoL; ACCESS, a case control etiologic study of sarcoidosis; ATS, American Thoracic Society; CASP, Critical Appraisal Skills Programme; ERS, European Respiratory Society; FSR-SARC, Foundation for Sarcoidosis Research—sarcoidosis advanced registry for cures; KSQ, King’s sarcoidosis questionnaire; OSAP, On-line Sarcoidosis Assessment Platform (accessible only from the USA); PROM, patient-reported outcome measure; ReAS, registry for advanced sarcoidosis; SAT, sarcoidosis assessment tool; SF-36, the medical outcome study 36-item short form health survey; SGRQ, St. George’s respiratory questionnaire; SHQ, sarcoidosis health questionnaire; WASOG, World Association of Sarcoidosis and Other Granulomatous Disorders; WHOQOL-BREF, World Health Organization quality of life-BREF assessment instrument.

a Data source: German Clinical Trials Register (DRKS00010072).

b At the most recent clinic visit (retrospective analysis).

c ClinicalTrials.gov NCT04342403.

d ClinicalTrials.gov NCT03769987.

**Table 2:** Clinical characteristics of the included studies and results related to the health-related quality of life

Study	Time since diagnosis (mean)	CXR stage (%)	Predicted DLCO (%)	Sarcoidosis medication (%)		Major outcomes related to cardiac sarcoidosis/HRQoL results
Bourbonnais *et al*. (2010)^38^	Not stated	0–8.6% I—18.1% II—41.5% III—14% IV—18.2%	61 ± 18	Steroidal IMD Steroidal + IMD	33.7% 42.3% 49.3%	**SF-36 scores (mean ± SEM)**: female (*n* = 157), male (*n* = 64) −Physical health: female 38 ± 1.8, male 47 ± 2.9 −Mental health: female 44 ± 1.75, male 52 ± 3.0 −Physical component summary: female 35 ± 0.8, male 38 ± 1.3 −Mental component summary: female 40 ± 0.8, male 43 ± 1.7 −SF-36 total score: female 42 ± 1.8, male 51.5 ± 3.1
						**SHQ scores (mean ± SEM):** female (*n* = 157), male (*n* = 64) −Daily functioning: female 3.8 ± 0.07, male 4.1 ± 0.2 −Physical functioning: female 3.4 ± 0.09, male 3.8 ± 0.13 −Emotional functioning: female 3.8 ± 0.08, male 4.1 ± 0.1 −SHQ total score: female 3.7 ± 0.07, male 4.2 ± 0.1
Bourbonnais *et al*. (2012)^39^	Not stated	0–8.2% I—17.6% II—40.9% III—15.1% IV—18.2%	62 ± 19	Steroidal IMD Steroidal + IMD	58% 42% 47%	**SF-36 scores (mean ± SEM):** −SF-36 total score: 45 ± 26 **SHQ scores (mean ± SEM):** −SHQ Total Score: 3.8 ± 1.1
Elfferich *et al*. (2011)^40^	5.1 years	0–I—44.9% II–IV—55.1%	86 ± 18	Prednisolone Pred + MTX	30.1% 29.9%	**WHOQOL-BREF (mean ± SD)** −Facet overall QoL: 11.9 ± 3.2 −Physical health: 12.5 ± 3.1 −Psychological health: 13.8 ± 2.5 −Social relationships: 15.0 ± 8.0 −Environment: 15.3 ± 2.5
Frye *et al*. (2021)^41^	>3 years for 77–84% of the cohort	Not stated	80	Not stated		**Mean baseline KSQ scores (IQR) (*P*-value, female vs. male)** for all participants (*n* = 200); female (*n* = 231) and males (*n* = 103) of the cohorts: −General health status (GHS): all 67.9 (51.9–80.2); female 69.8 (51.9–80.2) vs. male 64.2 (49.1–79.3) (*P* = 0.15) −Lung (L): all 77.8 (58.3–93.1); female 76.4 (58.3–93.1) vs. male 77.8 (58.3–93.1) (*P* = 0.88) −Medication (M): all 84.9 (54.6–100); female 84.9 (67.7–100) vs. male 77.3 (54.6–100) (*P* = 0.13) −Skin (S): all 88.2 (70.6–100); female 94.1 (70.6–100) vs. male 82.4 (70.6–100) (*P* = 0.15) −Eye (E): all 77.0 (52.7–93.2); female 77.0 (53.4–93.2) vs. male 73.0 (52.7–93.2) (*P* = 0.63) −GHS + L: all 73.0 (56.7–82.2); female 74.2 (59.1–84.0) vs. male 68.0 (53.4–80.9) (*P* = 0.28) −GHS + E: all 69.4 (54.4–80.6); female 71.7 (57.8–82.1) vs. male 68.3 (50.0–80.6) (*P* = 0.30) −GHS + skin (S): all 72.1 (60.0–83.6); female 75.4 (63.6–85.0) vs. male 67.9 (55.7–80.7) (*P* = 0.06) −GHS + L + S: all 73.1 (59.7–83.7); female 77.4 (62.3–85.4) vs. male 70.8 (50.8–81.6) (*P* = 0.15) −GHS + L + M: all 72.5 (59.2–82.0); female 76.8 (62.2–83.2) vs. male 69.4 (54.6–81.9) (*P* = 0.14) −GHS + S + M: all 72.3 (61.6–82.7); female 76.3 (67.6–84.4) vs. male 68.8 (57.8–79.2) (*P* = 0.02) −GHS + LH + SH + M: all 74.3 (60.7–83.3); female 78.4 (64.784.5) vs. male 71.8 (58.4–80.0) (*P* = 0.06)
Gvozdenovic *et al*. (2008)^42^	14.8 years	Not stated	Not stated	Not stated		**15D (mean ± SD) (range)**: 0.76 ± 0.16 (0.31–1.00)
						**SGRQ (mean ± SD) (range):** −Symptoms: 39.32 ± 24.03 (0–90.40) −Activities: 46.37 ± 26.32 (0–100) −Impacts: 31.73 ± 24.40 (0–93.02) −Total score: 37.43 ± 22.77 (0–90.51) **15D (mean ± SD) (range):** 0.76 ± 0.16 (0.31–1.00) **SGRQ (mean ± SD)**: total sample (*n* = 81); constituted by the exclusively pulmonary group (*n* = 49) and the pulmonary and extrapulmonary group (*n* = 32) −Symptoms: 39.32 ± 24.03; 37.32 ± 26.21 vs. 42.19 ± 20.56 −Activities: 46.37 ± 26.32; 40.17 ± 26.46 vs. 55.27 ± 23.77 −Impacts: 31.73 ± 24.40; 27.69 ± 23.83 vs. 37.55 ± 24.39 −Total: 37.43 ± 22.77; 33.07 ± 22.81 vs. 43.69 ± 21.55
Harper *et al*. (2020)^43^	11.7 years	Not stated	Not stated	Medications for sarcoidosis: 56% current 27% past 14% none 3% missing		**SHQ total score (mean ± SD)** for the total cohort (*n* = 2318) was 3.7 ± 1.1; reported by household income levels (*P*-value <0.001): −Income $0–34,999 (*n* = 577): 3.1 ± 1.0 −Income $35 000–84 999 (*n* = 795): 3.6 ± 1.0 −Income ≥$85 000 (*n* = 685): 4.1 ± 1.1
Judson *et al*. (2019)^44^	Not stated	0—52% I—14% II—9% III—6% IV—18%	Not stated	56% had sarcoidosis treatment ever 42% were on sarcoidosis treatment at the most recent follow-up		**SAT domain scores (mean ± SD)** of the cohort (*n* = 297): −Daily activities (*n* = 295): 45.3 ± 9.9 −Satisfaction (*n* = 295): 51.2 ± 11.5 −Pain (*n* = 295): 53.7 ± 11.1 −Sleep (*n* = 295): 53.2 ± 10.5 −Fatigue (*n* = 294): 54.3 ± 11.6 −Lung (*n* = 294): 42.9 ± 8.6
						SAT domain scores (mean ± SD) (*P*-value) for patients with symptoms (SP) and with no symptoms at presentation (NSP): −Daily activities: SP(*n* = 221) 44.4 ± 9.8; NSP(*n* = 74) 47.9 ± 9.5 (*P* = 0.007) −Satisfaction: SP(*n* = 222) 50.1 ± 11.6; NSP(*n* = 73) 54.4 ± 11 (*P* = 0.006) −Pain: SP(*n* = 219) 54.3 ± 11.2; NSP(*n* = 74) 52.0 ± 10.9 (*P* = 0.124) −Sleep: SP(*n* = 221) 53.6 ± 10.8; NSP(*n* = 74) 52.1 ± 9.2 (*P* = 0.297) −Fatigue: SP(*n* = 221) 55.1 ± 11.5; NSP(*n* = 74) 51.6 ± 11.4 (*P* = 0.022) −Lung: SP(*n* = 222) 43.2 ± 8.7; NSP(*n* = 72) 41.9 ± 8.3 (*P* = 0.267)
Judson *et al*. (2022)^45^	Not stated	0—8.4% I—3.0% II—6.9% III—5.1% IV—3.9% Missing—72.5%	Not stated	Any sarcoidosis therapy: 75.7% currently 17.2% in the past 4.0% never 3.1% missing		**KSQ scores**—not stated **SAT scores**—not stated **SGRQ scores**—not stated **PROMIS scores**—not stated
				Corticosteroid use: 48.3% currently 39.1% in the past 11.1% never 1.5% missing		
MihailovićVučinić *et al*. (2016)^46^	Not stated	0–I—67.3% II–IV—32.7%	Not stated	71.1% prednisolone only 13.3% prednisolone + MTX 6.1% MTX only 9.5% none		**SHQ scores (mean ± SD):** extrapulmonary group (*n* = 122) vs. exclusively pulmonary(*n* = 244); acute(*n* = 137) vs. chronic(*n* = 209) −Daily functioning: extrapulmonary 4.14 ± 0.89 vs. pulmonary 4.58 ± 0.90 (*P* < 0.001); acute 4.80 ± 0.87 vs. chronic 4.16 ± 0.86 (*P* < 0.001) −Physical functioning: extrapulmonary 4.45 ± 1.09 vs. pulmonary 4.77 ± 1.02 (*P* < 0.05); acute 5.00 ± 0.95 vs. chronic 4.43 ± 1.07 (*P* < 0.001) −Emotional functioning: extrapulmonary 4.12 ± 0.77 vs. pulmonary 4.26 ± 0.79 (*P* > 0.05); acute 4.35 ± 0.76 vs. chronic 4.13 ± 0.72 (*P* < 0.05) −SHQ total score: extrapulmonary 4.24 ± 0.77 vs. pulmonary 4.53 ± 0.76 (*P* < 0.001); acute 4.72 ± 0.72 vs. chronic 4.24 ± 0.75 (*P* < 0.001)
Obi *et al*. (2022)^47^	Not stated	0–39% I—13% II—19% III—13% IV—16%	76 ± 25	70% on any sarcoidosis medication Medications received: 40% prednisolone 20% MTX 19% Infliximab/adalimumab 10% hydrochloroquine 7% repository corticotropin 5% azathioprine 4% rituximab 3% leflunomide		**KSQ scores (mean ± SD)** (*P*-value): baseline (base) vs. 12-month follow-up (FU) for patients with advanced sarcoidosis: -General health status (GHS, *n* = 104): base 56 ± 17 vs. FU 59 ± 18 (*P* = 0.103) −Lung health (LH, *n* = 98): base 57 ± 18 vs. FU 60 ± 19 (*P* = 0.012) −Medication (*n* = 43): Base 43 ± 20 vs. FU 50 ± 20 (*P* = 0.034) −Skin health (*n* = 10): Base 25 ± 23 vs. FU 36 ± 23 (*P* = 0.058) −Eye health (*n* = 92): Base 62 ± 24 vs. FU 64 ± 24 (*P* = 0.272) −GHS + LH (*n* = 98): Base 59 ± 11 vs. FU 62 ± 13 (*P* = 0.004)
						**Baseline KSQ scores (mean ± SD)** (*P*-value) for all participants (All, *n* = 387); patients with advanced-sarcoidosis (Adv, *n* = 231); non-advanced-sarcoidosis (No-Adv, *n* = 156): −General health status (GHS, *n* = 384): All 62.50 ± 20.40; Adv 58.26 ± 18.75 vs. No-Adv 68.83 ± 21.16 (*P* < 0.001) −Lung health (LH, *n* = 381): All 63.78 ± 20.98; Adv 59.42 ± 19.40 vs. No-Adv 70.29 ± 21.61 (*P* < 0.001) −Medication (*n* = 153): All 49.82 ± 20.96; Adv 49.83 ± 21.69 vs. No-Adv 49.80 ± 18.92 (*P* = 0.993) −Skin health (*n* = 93): All 45.64 ± 29.29; Adv 42.77 ± 29.38 vs. No-Adv 50.85 ± 28.83 (*P* = 0.205) −Eye health (*n* = 374): All 68.16 ± 23.78; Adv 63.82 ± 24.42 vs. No-Adv 74.71 ± 21.25 (*P* < 0.001) −GHS + LH(*n* = 381): All 65.41 ± 15.48; Adv 61.77 ± 13.36 vs. No-Adv 70.83 ± 16.81 (*P* < 0.001)
						**Baseline SGRQ scores (mean ± SD)** (*P*-value): for all participants (All, *n* = 387); patients with advanced sarcoidosis (Adv, *n* = 231); non-advanced sarcoidosis (No-Adv, *n* = 156): −Symptoms (*n* = 367): All 45.61 ± 12.39; Adv 44.39 ± 13.44 vs. No-Adv 47.38 ± 10.44 (*P* = 0.017) −Activity (*n* = 368): All 26.75 ± 16.74; Adv 30.39 ± 17.63 vs. No-Adv 21.08 ± 13.48 (*P* < 0.001) −Impact (*n* = 381): All 39.99 ± 25.28; Adv 46.86 ± vs. No-Adv 29.77 ± 24.26 (*P* < 0.001) −Total (*n* = 348): All 36.99 ± 17.59; Adv 41.88 ± 16.79 vs. No-Adv 29.56 ± 19.17 (*P* < 0.001)
						**SGRQ scores (mean ± SD)** (*P*-value): baseline (Base) vs. 12-month follow-up (FU) for patients with advanced sarcoidosis: −Symptoms (*n* = 102): Base 45 ± 13 vs. FU 46 ± 16 (*P* = 0.478) −Activity (*n* = 97): Base 32 ± 17 vs. FU 34 ± 17 (*P* = 0.185) −Impact (*n* = 102): Base 48 ± 25 vs. FU 47 ± 25 (*P* = 0.602) −Total (*n* = 90): Base 43 ± 17 vs. FU 43 ± 18 (*P* = 0.969)
Tanizawa *et al*. (2019)^48^	9.1 years	0—31% I—26% II—28% III—12% IV—4%	85 ± 17	Not stated		SF-36, SHQ, and SGRQ scores for all enrolled at baseline (Base, *n* = 122) and baseline scores for those available at 5-year follow-up (BFU, *n* = 88): **SF-36 (mean ± SD):** −Physical functioning: Base 79.2 ± 20.9; BFU 80.5 ± 20.5 −Role-physical: Base 77.1 ± 27.0; BFU 79.2 ± 24.8 −Bodily pain: Base 71.9 ± 26.7; BFU 74.2 ± 24.4 −General health perceptions: Base 50.0 ± 19.7; BFU 49.5 ± 19.7 −Vitality: Base 52.2 ± 23.1; BFU 53.4 ± 22.3 −Social functioning: Base 78.6 ± 24.1; BFU 80.5 ± 22.7 −Role-emotional Base 77.7 ± 25.7; BFU 78.1 ± 25.7 −Mental health: Base 66.6 ± 20.5; BFU 67.1 ± 21.4
					
						**SHQ (mean ± SD):** −Daily functioning: Base 4.7 ± 1.0, BFU 4.7 ± 1.0 −Physical functioning: Base 5.4 ± 1.0, BFU 5.4 ± 0.9 −Emotional functioning: Base 4.8 ± 1.0, BFU 4.8 ± 1.0 −SHQ total score: 4.9 ± 0.9, BFU 4.9 ± 0.8
						**SGRQ (mean ± SD):** −Symptoms: Base 39.8 ± 20.6, BFU 39.1 ± 20.3
						−Activities: Base 29.7 ± 24.9, BFU 27.6 ± 23.9 −Impacts: Base 14.6 ± 14.6, BFU 13.6 ± 14.0 −Total score: 23.4 ± 16.3, BFU 22.1 ± 15.4

CXR, chest radiography; DLCO, diffusing capacity for carbon monoxide; HRQoL, health-related quality of life; IMD, immune modifying drugs; IQR, inter-quartile range; KSQ, King’s sarcoidosis questionnaire; MTX, methotrexate; SD, standard deviation; SEM, standard error of the mean; SF-36, the medical outcome study 36-item short form health survey; SGRQ, St. George’s respiratory questionnaire; SHQ, sarcoidosis health questionnaire; WHOQOL-BREF, World Health Organization Quality of Life-BREF assessment instrument.

The majority of studies was conducted in centres from the USA [*n* = 6 (55%)], with an equal proportion published in the last 3 years (2019–2022) (*Table 1*). The majority of samples was conducted in a single centre (*n* = 8, 73%), with just under half conducted between 2005 and 2009 (*n* = 5, 45%). None of the studies focused solely on CS patients or reported stratified HRQoL scores for CS patients, but four papers presented some adjusted analysis related to HRQoL and cardiac involvement.^41,45,46,48^ Almost half of studies (*n* = 5, 45%) used combinations of generic and sarcoidosis-specific HRQoL tools, and sarcoidosis-specific HRQoL were used individually in over a third of studies (*n* = 4, 36%). Only one study included longitudinal data for HRQoL assessments (follow-up every 12 months for 3 years) (*Table 1*).

Overall, 5334 sarcoidosis participants with mean age of 50 years were recruited across the 11 studies, including a total of 775 CS patients.

The average reported prevalence of CS was 14.5% (range Serbian^42^ 2% and German^41^ 3% to Japanese^48^ 22% and US citizen^43^ cohorts 21%^42^). Ethnicity was reported in seven studies,^38,39,43–47^ including Caucasian as the overall predominant ethnic background with considerable disparities between studies (range 9.5%^38^ to 100%^37^). Further socio-demographic and clinical characteristics are shown in the Supplementary material online, *Table S2*.

### Quality assessment and risk of bias

**Figure 2:**
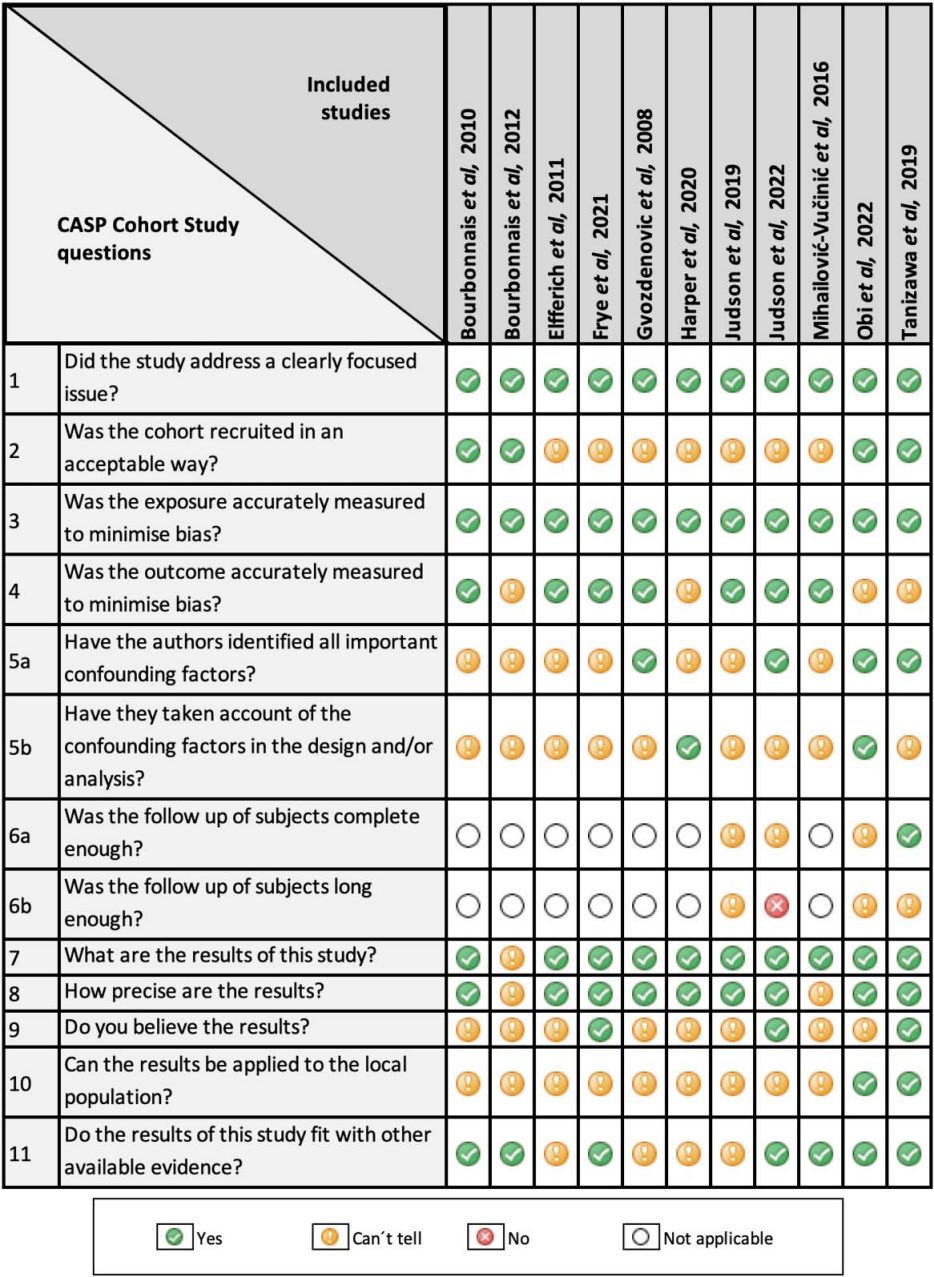
An illustration of the quality assessment (CASP checklist for cohort studies) for the 11 included studies. CASP, Critical Appraisal Skills Programme.

**Figure 3:**
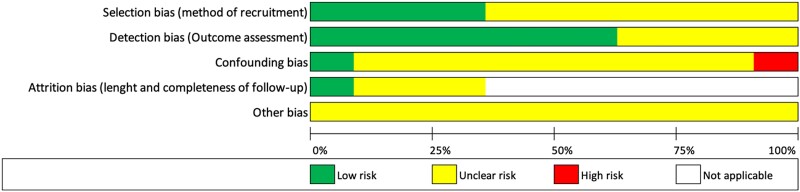
Risk of bias across the studies (*n* = 11). Eight of the 11 studies provided limited or no information about the method of recruitment; we considered these studies as unclear risk of selection bias. Four of the studies did not provide sufficient information to determine if the outcome was measured accurately and were deemed as unclear for detection bias. Based on a review of evidence and determined *a priori*, significant factors were identified to assess confounding bias. Three out of the four studies with follow-up did not provide sufficient information to determine completeness rates and were therefore deemed as unclear risk of attrition bias. Considering that none of the studies fulfilled the full CASP checklist, all the studies were marked as unclear risk for other bias. Additionally, the included studies did not provide sufficient socio-demographic characteristics. For example, three studies did not report the ethnicity of their samples,^40,42,48^ and only two studies provided data for the occupation and/or education level.^41,43^ Furthermore, the following reasons were noted to be deemed as unclear risk of other bias:
Limited CS representation (4–5%), socio-demographically imbalanced characteristics, scores for the SF-36 subscales not provided.^38,39^Limited CS representation (8%), no details were provided for the 25% of the sarcoidosis population who did not return study questionnaires.^40^Limited CS representation (3%), two references were used to describe the cohort of participants, but the provided samples were not matching with the reported data.^41^Limited CS representation (2%).^42^Online survey with no clinical characteristics. Self-reported socio-demographic characteristics. Only the SHQ total score was provided.^43^Retrospective analysis. Over half (363/660, 55%) of the cohort did not complete the SAT due to the validation date.^44^Monthly self-completed assessments using a new online platform for 6 months, and the study only reported baseline HRQoL scores.^45^Limited CS representation (7%). Only patients undergoing pulmonary function test during the clinical routine visits were eligible. No confidence intervals included.^46^Data analysed retrospectively from a multi-national database (*n* = 456). Participants with advanced sarcoidosis (*n* = 231) were followed up every 6 months for 3 years, but only baseline HRQoL assessments were reported. The analysis included over one-half (53%) of the cohort with advanced sarcoidosis and completed a 12-month visit.^47^Participants completed a follow-up 5-year visit, but HRQoL assessments were included only at baseline.^48^Other bias was deemed as unclear risk for all the studies considering that none of the studies fulfilled the full CASP checklist for cohort studies as detailed in *Figure 2*.

The percentage scores of CASP checklists and the appraised levels of evidence are presented in *Table 1*. Over half of the included studies (*n* = 6, 54%) were categorized as poor quality and the remaining publications (*n* = 5, 46%) as moderate quality. *Figure 2* illustrates the quality assessment for each of the included studies. None of the studies fulfilled the full CASP checklist for cohort studies. Considering study designs, cross-sectional studies and retrospective studies scored lower compared with observational studies. The poorest-rated section was the adequacy of follow-up of cohorts, which met the criteria only in one study. Furthermore, only 18% (*n* = 2) of the studies met the ‘consideration of confounding factors in the design’ and ‘application of results to local population’. *Figure 3* demonstrates the predominant ‘unclear risk of bias’ due to the lack of information provided across all the manuscripts.

### HRQoL outcome by measurement tool

There was a lack of standardization in the HRQoL tools used across studies. The majority of the studies (*n* = 9, 81%) included sarcoidosis-specific HRQoL PROMs: the sarcoidosis health questionnaire (SHQ) was used in five (45%), the King’s sarcoidosis questionnaire (KSQ) in three (27%), and the sarcoidosis assessment tool (SAT) in two (18%). In addition, some studies were complemented by a generic HRQoL PROM such as the 36-item short form (SF-36) (*n* = 3, 27%) and/or the respiratory-specific HRQoL PROM St. George’s respiratory questionnaire (SGRQ) (*n* = 3, 27%).

### HRQoL measured by the sarcoidosis health questionnaire

Among the two studies that solely used the SHQ,^43,46^ the Serbian study found that patients with multi-organ involvement had the lowest mean SHQ scores for daily functioning (3.72 ± 0.91), physical functioning (4.00 ± 0.98), and emotional functioning (3.74 ± 0.95).^46^ The CS patients (*n* = 23, 7%) had lower scores for physical functioning (ANOVA *F* = 1.523, *P* = 0.041, SHQ scores were not provided). Moreover, patients receiving monotherapy with methotrexate (MTX, *n* = 21, 6%) had better SHQ scores (*P* < 0.05 for all domains) in comparison with those with combined therapy (MTX + prednisolone, *n* = 46, 13%).^46^ Three studies combined the SHQ with SF-36.^38,39,43^ Statistically significant better HRQoL was reported in men according to the SHQ physical functioning and SHQ total.^38^ The Japanese study^48^ showed that 23% (*n* = 20) of those who completed follow-up investigations (*n* = 88/122) had a 5-year deterioration, which was significantly associated with the SHQ total (adjusted OR 0.54, 0.29–0.99) and the SHQ domain physical functioning scores (OR 0.48, 0.27–0.87) after adjusted for CS at enrolment.

### HRQoL measured by the King’s sarcoidosis questionnaire

The KSQ was used solely in a German cohort^41^ (*n* = 200) reporting that patients with multiple organ involvement had significantly lower scores for the KSQ subdomain general health status (GHS) (adjusted R2 = 0.56, *P* < 0.001) and those receiving sarcoidosis-specific therapy had worse HRQoL (*P* < 0.002).^41^ Cardiac involvement lowered the GHS score by estimate 4.25 points (95% CI −21.20 to 12.70, *P* = 0.621).^41^ Among the studies combining the KSQ with additional HRQoL tools, worse dyspnoea was associated with lower scores for all the KSQ subdomains (*P* < 0.001), including the strongest correlations for the GHS (rho 0.635) and lung health (LH) (rho 0.724).^47^ Similarly, fatigue was strongly correlated with the GHS + LH (rho −0.800).^47^ Lower baseline modified extrapulmonary organ assessment tool (MePOST of <3), which incorporated both the number of organs and the severity of organs involved, was associated with statistically better outcomes for 9 of the 15 outcome of interest for sarcoidosis patients (OIPs): unscheduled healthcare visits, workdays missed, steps walked, prednisolone dose, pain, satisfaction, sleep, lung concerns, and global health perception.^45^

### HRQoL measured by the sarcoidosis assessment tool

The presence of symptoms at the initial visit was statistically and clinically associated with worse HRQoL (*P* < 0.022 for the SAT modules daily activities, satisfaction, and fatigue), significantly more organ involvement, and more frequent requirement for any sarcoidosis-specific therapy (*P* < 0.001).^44^ Moreover, cardiac involvement at baseline was associated with 3 of the 15 selected OIPs including higher doses of prednisolone (*P* < 0.0001), more consumed calories (*P* < 0.0027), and more sleep disturbance based on the SAT subdomain (*P* < 0.0336) after a 6-month follow-up.^45^

### HRQoL measured by the patient visual analogue scale

Less than 7 cm (out of a total of 10 cm) on the patient visual analogue scale (patient-VAS) at baseline was statistically associated with worse outcomes in 13 of the 15 selected OIPs (all except steps walked and calories consumed), which was superior to the additional physiologic, radiographic, or HRQoL measures.^45^

### HRQoL measured by SF-36

Among the three studies using SF-36 in combination with the SHQ,^38,39,48^ two studies reported limited SF-36 results using the total^38,39^ and the physical and mental component^38^ scores. Their conclusions were that men had better HRQoL (*P* < 0.02 for SF-36 scores)^38^ and the SF-36 was a valid HRQoL tool for sarcoidosis.^39^ Moreover, three SF-36 domains (general health perception, vitality/energy, and limitations in usual role activities due to physical health problems) had statistically significant associations with a 5-year clinical deterioration, but only baseline HRQoL scores were collected to evaluate the long-term deterioration.^48^

### HRQoL by WHOQOL-BREF

The sarcoidosis group had statistically significant better HRQoL compared with the idiopathic pulmonary fibrosis group, including the WHOQoL-BREF domains ‘environment’ (*P* < 0.05), ‘physical health’ (*P* < 0.01), and ‘facet overall QoL’ (*P* < 0.01) (see *Table 2*). Moreover, the prevalence of type D or ‘distressed’ personality in both groups did not differ from a general population.^40^

### HRQoL measured by the 15-dimensional measure of HRQoL

The 15-dimensional (15D) was combined with the SGRQ, but there was not any significant difference for the 15D results when segregated by ‘isolated pulmonary’ or ‘pulmonary plus extrapulmonary’ sarcoidosis cases (*P* = 0.106).^42^

### Factors influencing HRQoL in cardiac sarcoidosis patients

The 11 identified studies reported various factors influencing HRQoL in sarcoidosis cohorts, with variable CS representation. These associations include potential predictors of HRQoL in CS patients related to socio-demographic, symptomatology, therapy, physiologic, and clinical status (*Table 3* and Supplementary material online, *Table S3*).

**Table 3:** Factors impacting on HRQoL in studies with CS representation

Associated predictor/factor/parameter	Impact on HRQoL^a^	HRQOL tools used	Ref
**Socio-demographic**			
Female gender	Worsens	SHQ	^43,46^
Patients within 41–50 years old	Worsens	SHQ	^46^
Younger age	Worsens	SHQ	^43^
Reduced household income (self-reported <$35 000/year)			
Larger household size			
**Sarcoidosis-specific**			
‘Chronic’ sarcoidosis	Worsens	SHQ	^46^
Multi-organ sarcoidosis involvement	Worsens	SHQ, SGRQ	^42,46^
Extrapulmonary sarcoidosis involvement	Worsens	SHQ	^46^
Development of sarcoidosis-associated	Worsens	SHQ	^43^
Presence of symptoms at the initial visit	Worsens	SAT	^44^
**Specific symptoms**			
Depression	Worsens	WHOQOL-BREF	^40^
Fatigue			
Type D or ‘distressed’ personality	Worsens	WHOQOL-BREF	^40^
Dyspnoea/lower baseline dyspnoea index (BDI)	Worsens	KSQ, SGRQ	^47^
Cardiac sarcoidosis involvement	Worsens	SHQ	^46,48^
**Therapy-related factors**			
Use of monotherapy with corticosteroids (CS) or immunosuppressant sparing regimen (IS) vs. use of combined therapy (CS + IS)	Improves	SHQ	^46^
CS + IS in chronic sarcoidosis	Worsens	SHQ	^46^
Systemic CS/IS usage at enrolment	Worsens	SHQ	^48^
Past/current use of oral sarcoidosis therapy	Worsens	SHQ	^43^
Development of steroid-associated comorbidities			
**Physiologic and clinical parameters**			
BDSS at 6 min	Worsens	SF-36	^38,39^
Reduced DSP	Worsens	SF-36	^39^
Reduced 6MWD	Worsens	SF-36, SHQ	^38,39,45^
6MWD ≥ 420 m	Improves		
FVC ≥ 80% predicted	Improves	SAT, PROMIS	^45^
FEV1 < 80% predicted	Worsens		
Low DLCO values	Worsens	SF-36	^38^
Decreased LVEF at baseline	Worsens	SHQ	^48^
**PROM scale/subscale**			
SF-36 vitality subscale	Long-term clinical deterioration	SF-36	^48^
SF-36 physical domain			
SF-36 bodily pain			
SHQ physical functioning	Long-term clinical deterioration	SHQ	^48^
SHQ daily functioning after adjusting for cardiac involvement at enrolment			

6MWD, 6-min walking distance; BDSS, Borg dyspnoea scale score; CS, corticosteroid; DLCO, diffusing capacity for carbon monoxide; DSP, distance–saturation product; FVC, forced vital capacity; FEV1, forced expiratory volume in 1 s; HRQoL, health-related quality of life; IS, immunosuppressant sparing regimen; KSQ, King’s sarcoidosis questionnaire; LVEF, left ventricular ejection fraction; m, metre; min, minute; PROMIS, patient-reported outcomes measurement information system; ref, reference; SF-36, the medical outcome study 36-item short form health survey; SGRQ, St. George’s respiratory questionnaire; SHQ, sarcoidosis health questionnaire; WHOQOL-BREF, World Health Organization Quality of Life-BREF assessment instrument.^48^

a Data supporting these associations from the original publications are reported in Supplementary material online, *Table S3*.

## Discussion

In 2011, the World Association of Sarcoidosis and other Granulomas disease (WASOG)^56^ recommended that all research studies should incorporate HRQoL measurement; however, evidence remains limited.^24,57,58^ This review summarizes 11 studies that included CS patients exploring HRQoL as primary or secondary outcome, of which only 6 included data collected since the WASOG recommendation. Furthermore, the three current clinical diagnostic tools for CS were introduced between 2014 and 2017.^14,52,59^ Despite five studies collected data between 2014 and 2020, only two^45,47^ used one of the latest tools (WASOG, 2014)^52^ which could have limited the accuracy to define cardiac involvement in the included sarcoidosis cohorts (*Table 1*).^49^ Our findings potentially demonstrate impaired HRQoL in patients with CS; however, data need to be interpreted with caution because of the lack of CS-specific studies, the lack of disease-specific standardized HRQoL tools, and the appraised low to moderate evidence levels. For instance, the higher incidence of CS in Japanese cohorts^60^ or the lack of longitudinal assessments to measure changes in HRQoL^61^ should be considered when interpreting that certain baseline HRQoL scores (SHQ and SF-36) were associated with a 5-year clinical deterioration.^48^ Also, sarcoidosis patients with ‘extrapulmonary’^42,46^ or ‘advanced disease’^47^ had worse HRQoL, including CS patients in these impaired groups. However, the generalizability of these findings is limited by the small number of CS patients in these cohorts (range 2–17%).^42,46,47^

The majority of the studies (*n* = 10, 91%) reported HRQoL results for a single timepoint (baseline), and only two studies (18%) included longitudinal HRQoL assessments at 6 months^45^ and every 12 months^47^ up to 3 years. This is unsatisfactory to determine the impact of a complex disease and HRQoL impairment over time. Previous work has recommended to initiate systemic sarcoidosis therapy in CS,^27^ which is usually combined with cardiac-specific treatments and likely to impact on HRQoL at any specific time. Similarly, a *post hoc* exploratory analysis of HRQoL in sarcoidosis outpatients with corticosteroid therapy suggested that CS patients had a greater impact on HRQoL compared with patients with non-cardiac involvement.^32^ Therefore, there is a need to incorporate longitudinal ‘snapshot’ HRQoL assessments and to describe HRQoL scores thoroughly for patients with cardiac and extracardiac involvement.

Overall, we also identified that there was no consistency in the HRQoL tools used, with contradictory conclusions regarding the relevance of generic and/or sarcoidosis-specific HRQoL tools.^57^ Furthermore, these sarcoidosis-specific tools do not include any cardiac-specific subscale/item. Certainly, the development of CS-specific items/tools and/or adaptation of existing sarcoidosis-specific PROMs would be a substantial advance for clinical research and to understand the impact of CS.^23^ Indeed, the implementation of these HRQoL tools in clinical practice will help to maximize the quality of care considering the indications for systemic therapy and the need to evaluate HRQoL in CS patients.^21,62,63^

A recent study focussing on sarcoidosis healthcare costs found that CS patients had higher healthcare costs compared with those without cardiac involvement.^64^ In the same way, CS was reported as a predictor for hospitalizations, development of new sarcoidosis-related comorbidities, and severe impairment on family finances.^43^ Therefore, there is an opportunity to quantify the CS-associated costs in detail, assessing the CS-related impact on work ability and productivity, and the burden for disability claims which may impact on HRQoL.^15^

Many unmet and urgent needs remain for people with CS. This review provides potential factors impacting on HRQoL based on sarcoidosis cohorts with CS representation. For instance, one study supported psychological screening, and if needed, the incorporation of psychological counselling, coping strategies, and emotional support in the management of sarcoidosis.^40^ Similarly, recent reviews have suggested potential factors influencing HRQoL^58^ and sarcoidosis-related manifestations to be considered during disability evaluations.^15^ For example, stress or anxiety may contribute to palpitations. Thus, multidisciplinary CS teams should ensure comprehensive care and holistic management considering individual needs and concerns.^65^

## Limitations

This review has three main limitations. Firstly, none of the included studies reported specific HRQoL results for CS patients, and only 4 of the 11 presented some adjusted HRQoL results for sarcoidosis patients with cardiac involvement. Therefore, the interpretation of these HRQoL findings and the generalizability is challenging. Secondly, no studies were excluded on the basis of quality assessment or risk of bias. This was to enable full exploration of insightful studies with multi-organ manifestations. However, the data availability bias could have contributed to mask other potential bias, such as selection bias (population) or reporting bias. Thirdly, due to the heterogeneity of the outcome measures and variations in the original designs, this review had limited data to conduct direct comparisons or meta-analysis. Despite these limitations, this methodological robust review is valuable in describing and summarizing the current HRQoL evidence for sarcoidosis cohorts with cardiac involvement representation.

## Conclusions

In conclusion, patients with CS are included in sarcoidosis cohorts with impaired HRQoL and worse clinical outcomes. However, sarcoidosis studies with HRQoL as primary or secondary outcome do not incorporate stratified HRQoL scores for patients with cardiac involvement. There is a need for further research, especially for longitudinal and multicentre studies, using complementary HRQoL tools to assess CS cohorts. The development of CS-specific and/or adaptation of existing sarcoidosis tools would be a substantial advance to understand and evaluate the impact of CS on HRQoL, improving the quality of care for a complex disease.

## Supplementary Material

oead009_Supplementary_Data

## Data Availability

The data underlying this article are available in the article and in its online supplementary material. Any additional data will be shared on reasonable request to the corresponding author.
