# Supplementary material for: Health-related quality of life in cardiac sarcoidosis: a systematic review
Source: Eur Heart J Open. 2023 Feb 18;3(2):oead009. doi: 10.1093/ehjopen/oead009 (PMC10039618; doi:10.1093/ehjopen/oead009)
Supplement: oead009_Supplementary_Data [file oead009_supplementary_data.pdf]

Table S1   Search syntax adapted for electronic databases

#	Database	Search term
**1**	Medline	(exp SARCOIDOSIS/ AND cardiac) OR (cardiac ADJ3 sarcoid*) OR (heart ADJ3 sarcoid*).ti,ab
**2**	Medline	exp "QUALITY OF LIFE"/ OR exp FATIGUE/ OR ("quality of life" OR qol OR "health status" OR wellbeing OR satisfaction OR improvement* OR fatigue OR energy OR tiredness OR exhaustion OR anxi* OR burden OR exercise OR cognitive OR depressi* OR driv* OR pain* OR panic OR rehab*).ti,ab
**3**	Medline	(1 AND 2)
**4**	EMBASE	"CARDIAC SARCOIDOSIS"/ OR (cardiac ADJ3 sarcoid*) OR (heart ADJ3 sarcoid*).ti,ab
**5**	EMBASE	exp "QUALITY OF LIFE"/ OR exp FATIGUE/ OR ("quality of life" OR qol OR "health status" OR wellbeing OR satisfaction OR improvement* OR fatigue OR energy OR tiredness OR exhaustion OR anxi* OR burden OR exercise OR cognitive OR depressi* OR driv* OR pain* OR panic OR rehab*).ti,ab
**6**	EMBASE	(4 AND 5)
**7**	CINAHL	"cardiac sarcoidosis" OR (cardiac ADJ3 sarcoid*) OR (heart ADJ3 sarcoid*).ti,ab
**8**	CINAHL	exp "QUALITY OF LIFE"/ OR exp FATIGUE/ OR ("quality of life" OR qol OR "health status" OR wellbeing OR satisfaction OR improvement* OR fatigue OR energy OR tiredness OR exhaustion OR anxi* OR burden OR exercise OR cognitive OR depressi* OR driv* OR pain* OR panic OR rehab*).ti,ab
**9**	CINAHL	(7 AND 8)
**10**	BNI	"cardiac sarcoidosis" OR (cardiac ADJ3 sarcoid*) OR (heart ADJ3 sarcoid*).ti,ab
**11**	PsycINFO	"cardiac sarcoidosis" OR (cardiac ADJ3 sarcoid*) OR (heart ADJ3 sarcoid*).ti,ab
**12**	AMED	"cardiac sarcoidosis" OR (cardiac ADJ3 sarcoid*) OR (heart ADJ3 sarcoid*).ti,ab

Table S2 Participant baseline sociodemographic characteristics from included studies

	Mean age (SD)	Female (%)	Ethnicity (%)	Level of education (%)	Household income (%)	Employment status (%)
Bourbonnais *et al.* (2010)[38]	40 ± 10.6	71%	90.5% African American<br>9.5% White	—	—	—
Bourbonnais *et al.* (2012)[39]	45.9 ± 11.1	72%	86.8% African American<br>13.2% White	—	—	—
Elfferich *et al.* (2011)[40]	45.4 ± 11.4	45.8%	—	—	—	—
Frye *et al.* (2021)[41]	53.3 ± 12.7	48.5%	—	28.9% Elementary school<br>33% Secondary school<br>1% Polytechnic secondary school<br>7.2% Technical colleague qualification<br>22.7% University qualification<br>3.6% Other or No certificate	—	60.8% Employed<br>30.4% Unemployed
Gvozdenovic *et al.* (2008)[42]	48.2 ± 11.3	75.3%	—	—	—	—
Harper *et al.* (2020)[43]	52.2 ± 10.7	74.1%	18% African American<br>73% White<br>8% Other<br>1% Missing	20% >college<br>29% college<br>28% >high school (HS) no college degree<br>16% HS or less<br>7% missing	30% >$85,000<br>34% $35,000-$84,999<br>25% $0K-$34,999<br>11% missing	29% having lost their job due to their sarcoidosis

Judson *et al.* (2019)[44]	56 ± 13.6	55%	79 % White<br>19.5% Black<br>10 % Other	—	—	—
Judson *et al.* (2022)[45]	54.7 ± 10.8	64.5%	62.8% White<br>26.8% Black<br>4.1% Other<br>6.2% Missing	—	—	—
Mihailović-Vučinić *et al.* (2016)[46]	46 ± 10.8	74.3%	100% White	—	—	—
Obi *et al.* (2022)[47]	55.8 ± 10.0	60%	61.8% Caucasian<br>34.6% African American<br>3.7% Others	—	—	—
Tanizawa *et al.* (2019)[48]	56.8 ± 15.5	60.6%	—	—	—	—

Table S3 Factors impacting on HRQoL in studies with CS-representation

Associated Factor/ predictor/ parameter	Impact on HRQoL	HRQOL-PROMs used	Data extracted from the published manuscripts including supplementary materials	Ref
**Socio-demographic**				
Female gender	Worsens	SHQ	In a multivariate linear regression, female gender was an independent predictor for lower SHQ total (β=-0.138, *P* <0.010) and emotional functioning (β=-0.165, *P* <0.003) scores[46]	46
			In a multivariate linear regression, female gender was a predictor of lower SHQ total score (*P* <0.001). When plotted in a random forest modelling, female gender was the ninth highest impact variable on SHQ score[43]	43
Patients within 41-50 years old	Worsens	SHQ	Patients in the 41-50y age group had the lowest SHQ total score (4.25 ± 0.76), followed by the 51-60y (n=95, 4.31 ± 0.75) and the 61-70y (n=27, 4.34 ± 0.59) groups[46]	46
Younger age	Worsens	SHQ	In a multivariate linear regression, these factors were predictors of lower SHQ total score (*P* <0.001). When plotted in a random forest modelling, younger age was the highest impact variable on SHQ score, followed by lower income (third), larger household size (fourth)[43]	43
Reduced household-income (self-reported <$35,000/year)				
Larger household size				
**Sarcoidosis-specific**				
"Chronic" sarcoidosis	Worsens	SHQ	In a multivariate linear regression, chronic disease was an independent predictor for lower SHQ total (β=-0.216, *P* <0.000), daily functioning (β=-0.0.449, *P* <0.004) and physical functioning (β=-0.202, *P* <0.000) scores[46]	46
Multi-organ (three or more) sarcoidosis involvement	Worsens	SHQ	Patients in whom the sarcoidosis affected three or more organs had the lowest mean total SHQ score (3.82 ± 0.94), as well as the lowest mean scores for the domains daily functioning (3.72 ± 0.91), physical functioning (4.00 ± 0.98), and emotional functioning (3.74 ± 0.95)[46]	46
		SGRQ	Differences for SGRQ symptoms (*P* <0.040) and total (*P* <0.010) scores [42]	42
Extrapulmonary sarcoidosis involvement	Worsens	SHQ	Patients with extrapulmonary involvement had lower scores for daily functioning (*P* <0.001), physical functioning (*P* <0.005) and Total (*P* <0.001)	46

Development of sarcoidosis-associated	Worsens	SHQ	In a multivariate linear regression, it was predictor of lower SHQ total score (*P* <0.001). When plotted in a random forest modelling, this was the second highest impact variable on SHQ score[43]	43
Presence of symptoms at the initial visit	Worsens	SAT	Worse HRQoL on the SAT domains daily activities (*P* <0.007), satisfaction (*P* <0.006) and fatigue (*P* <0.022)[44]	44
**Specific symptoms**				
Depression<br>Fatigue	Worsens	WHOQOL-Bref	Depressive symptoms (β=-0.129) and fatigue predicted poor overall HRQoL (F=187.0, *P* <0.001)[40]	40
Type-D or "distressed" personality	Worsens	WHOQOL-Bref	Type-D sarcoidosis patients had lower HRQoL scores (t=4.20, *P* <0.001)[40]	40
Dyspnoea / Lower Baseline Dyspnoea Index (BDI)	Worsens	KSQ, SGRQ	Lower BDI total scores were correlated to worse HRQoL (all *P* <0.001): KSQ-General Health status (rho 0.635), KSQ-Lung health (rho 0.724), KSQ-Medication (rho 0.318), KSQ-Skin Health (rho 0.353), KSQ-Eye Health (rho -0.450), SGRQ-activity (rho -0.623), SGRQ-impact (rho -0.794), SGRQ-total (rho -0.785)[47]	47
Cardiac sarcoidosis involvement	Worsens	SHQ	Patients with cardiac sarcoidosis had lower SHQ scores for the physical functioning (ANOVA F = 1.523, p = 0.041) domain[46]. In a multivariate linear regression, cardiac disease was an independent predictor for lower SHQ daily functioning (β=0.345, *P* <0.004) scores[46]	46
			In multivariate logistic regression, cardiac involvement was associated for 5-year clinical deterioration with the SHQ-physical functioning (OR= 3.10, [95%CI 1.03-9.37], *P*=0.045) and SHQ-total (OR= 2.95, [95%CI 0.99-8.81], *P*=0.053) scores[48]	48
**Therapy related factors**				

Use of monotherapy with corticosteroids (CS) or corticosteroid sparing regimen (IS), versus use of combined therapy (CS+IS)	Improves	SHQ	SHQ scores (mean ± SD) [ANOVA, *P*-value] by treatment regimen[46]:<br>none (n=33); Prednisone only (n=246); MTX only (n=21); MTX+Pred (n=46)<br>-Daily functioning: none 4.71 ± 0.94; Pred 4.51 ± 0.93; MTX 4.15 ± 0.64; MTX+Pred 3.86 ± 0.68 [F 5.49, *P*<0.0001]<br>-Physical functioning: none 4.98 ± 1.11; Pred 4.78 ± 1.01; MTX 4.05 ± 0.96; MTX+Pred 4.05 ± 1.04 [F 6.11, *P*<0.0001]<br>-Emotional functioning: none 4.33 ± 0.88; Pred 4.28 ± 0.79; MTX 3.93 ± 0.74; MTX+Pred 3.93 ± 0.66 [F 3.27, *P*<0.05]<br>-SHQ Total Score: none 4.67 ± 0.82; Pred 4.52 ± 0.76; MTX 4.05 ± 0.58 MTX+Pred 3.95 ± 0.67 [F 6.68, *P*<0.0001]	46
CS+IS in chronic sarcoidosis	Worsens	SHQ	In a multivariate linear regression, chronic sarcoidosis patients treated with the methotrexate-prednisone combination had lower scores for the SHQ domains physical functioning (β=-0.153, *P* <0.008) and emotional functioning (β=0.120, *P* <0.003), as well as lower total SHQ scores (β=0.143, *P* <0.013), in comparison with chronic sarcoidosis patients on prednisolone alone[46]	46
Systemic CS/IS usage at enrolment	Worsens	SHQ	SHQ total score had a weak association for long-term deterioration with systemic CS/IS usage at enrolment (rho= -0.23, *P*=0.03)[48]	48
Past/current use of oral sarcoidosis-therapy	Worsens	SHQ	In a multivariate linear regression, these were predictors of lower SHQ total score (*P* <0.001). When plotted in a random forest modelling, “current or past medication use” was the fifth and “new steroid comorbidity” was the eight highest impact variable on SHQ score[43]	43
Development of steroid-associated comorbidities				
**Physiologic and clinical parameters**				
BDSS at 6-min	Worsens	SF-36	In a multivariate linear regression, the BDSS at six minutes was associated with poor HRQoL scores (β=-3.4 *P* <0.001)[38]	38
			In a multivariate linear regression, the BDDS at six minutes was associated with poor HRQoL scores [95% CI: -4.3 – (-0.16), *P* <0.04][38]	39
Reduced DSP	Worsens	SF-36	In a multivariate linear regression, the DSP was associated with poor HRQoL scores: SF-36 [95% CI: 0.082 – 0.02), *P* <0.001] and SHQ [95% CI: 0.002 – 0.001), *P* <0.009][38]	39
		SHQ		

Reduced 6MWD	Worsens	SF-36,	In a multivariate linear regression, reduced 6MWD was associated with poor HRQoL scores (β=0.4 *P* <0.003)[38]	38
		SF-36, SHQ	In a multivariate linear regression, reduced 6MWD was associated with poor HRQoL scores: SF-36 [95% CI: 0.031 – 0.1), *P* <0.01] and SHQ [95% CI: 0.01 – 0.04), *P* <0.01][38]	39
6MWD ≥420m	Improves	SAT, PROMIS	Statistically significant associations with ten of 15 outcomes including: steps, calories, missed workdays, satisfaction, pain, sleep disturbance, fatigue, lung concern, depression, and global health[45]	45
FVC ≥80% predicted	Improves		Statistically significant associations with seven of 15 outcomes including: steps, calories, sleep disturbance, lung concern, global health, hospitalisations, and unscheduled clinic visits[45]	
FEV1 <80% predicted	Worsens		Statistically significant associations with four of 15 outcomes including: pain, sleep disturbance, lung concern and global health[45]	
Low DLCO values	Worsens	SF-36	In a multivariate linear regression, the DLCO was associated with poor HRQoL scores (β=-1.46 *P* <0.001)[38]	38
Decreased LVEF at baseline	Worsens	SHQ	In multivariate logistic regression, decreased LVEF at enrolment had a marginal correlation for long-term deterioration with the SHQ total score (rho= 0.19, *P*=0.07)[48]	48
**PROM scale/subscale**				
SF-36 Vitality subscale	Long-term clinical deterioration	SF-36	Univariate logistic regression for 5-year clinical deterioration[48]:<br>-Vitality subscale OR 0.97 [95%CI 0.94-0.99], *P*=0.01<br>-Bodily pain subscale OR 0.98 [95%CI 0.96-1.00], *P*=0.05	48
SF-35-Bodily pain subscale				
SF-36-Physical domain			Two of the six questions related to pain (headache and arthralgia) were associated with 5-year clinical deterioration (unadjusted OR 0.79, [95%CI, 0.58–0.89])[48]	
SHQ-Physical functioning after adjusting for cardiac involvement at enrolment	Long-term clinical	SHQ	In multivariate logistic regression, SHQ-physical functioning was associated for 5-year clinical deterioration (rho= 0.48, *P*=0.02)[48]	48

SHQ-Daily Functioning after adjusting for cardiac involvement at enrolment	deterioration		In multivariate logistic regression, SHQ-daily functioning was associated for 5-year clinical deterioration (adjusted OR= 0.59 [0.34-1.00])[48]	

Abbreviations: 6MWD, 6-minutes walking distance; β, beta; ANOVA, analysis of variance; BDSS, borg dyspnea scale score; CS, corticosteroid, DLCO, diffusing capacity for carbon monoxide; DSP, distance-saturation product; FVC, forced vital capacity; FEV1, Forced expiratory volume in 1 second; HRQoL, health-related quality of life; IS, steroid sparing immunosuppressants; KSQ, king's sarcoidosis questionnaire; LVEF, left ventricular ejection fraction; m, meter; min, minute; MTX, methotrexate; OR, odds ratio; *P*, P-value; PROMIS, Patient-Reported Outcomes Measurement Information System; ref, reference; rho, Spearman's rank correlation coefficient; SD, standard deviation; SF-36, The Medical Outcome Study 36-item Short Form Health Survey; SGRQ, st.george's respiratory questionnaire, SHQ, sarcoidosis health questionnaire; t, Student's t-test; WHOQOL-bref, World Health Organization Quality of Life-BREF assessment instrument; y, years.
